# Unravelling the molecular network structure of biohybrid hydrogels

**DOI:** 10.1016/j.mtbio.2025.102249

**Published:** 2025-08-26

**Authors:** Jana Sievers-Liebschner, Ron Dockhorn, Jens Friedrichs, Thomas Kurth, Peter Fratzl, Jens-Uwe Sommer, Carsten Werner, Uwe Freudenberg

**Affiliations:** a Leibniz Institute of Polymer Research Dresden, Division Polymer Biomaterials Science, Max Bergmann Center of Biomaterials Dresden, 01069, Dresden, Germany; b Technische Universität Dresden, Center of Regenerative Therapies Dresden and Cluster of Excellence Physics of Life, 01069, Dresden, Germany; c Leibniz Institute of Polymer Research Dresden, Division Theory of Polymers, Dresden, 01069, Dresden, Germany; d Technische Universität Dresden, Institute for Theoretical Physics and Cluster of Excellence Physics of Life, 01069, Dresden, Germany; e Technische Universität Dresden, Center for Molecular and Cellular Bioengineering, Technology Platform Core Facility Electron Microscopy and Histology, 01307, Dresden, Germany; f Max Planck Institute of Colloids and Interfaces, Department of Biomaterials, 14476, Potsdam, Germany

**Keywords:** Hydrogel, Glycosaminoglycan, Nanoscale polymer network, Transmission electron microscopy, X-ray scattering, Computer simulation

## Abstract

Glycosaminoglycan-based biohybrid hydrogels represent a powerful class of cell-instructive materials with proven potential in tissue engineering and regenerative medicine. Their biomedical functionality relies on a nanoscale polymer network that standard microscopy techniques cannot resolve. Here, we introduce an advanced analytical approach that integrates transmission electron microscopy, X-ray scattering, and computer simulations to directly and quantitatively characterize the nanoscale molecular network structure of these hydrogels. This method provides detailed insights into network connectivity and inhomogeneities, which are critical factors for understanding their functional properties and the cell-instructive cues they determine. Given that the glycosaminoglycan-based hydrogels facilitate the controlled delivery of soluble growth factors and guide the growth of complex organoid cultures, our approach also illuminates essential aspects of cell-material interactions and remodeling processes. Ultimately, this integrated strategy enables the precise customization of engineered matrices for regenerative therapies and disease/tissue modeling.

## Graphical abstract

**Figure:**
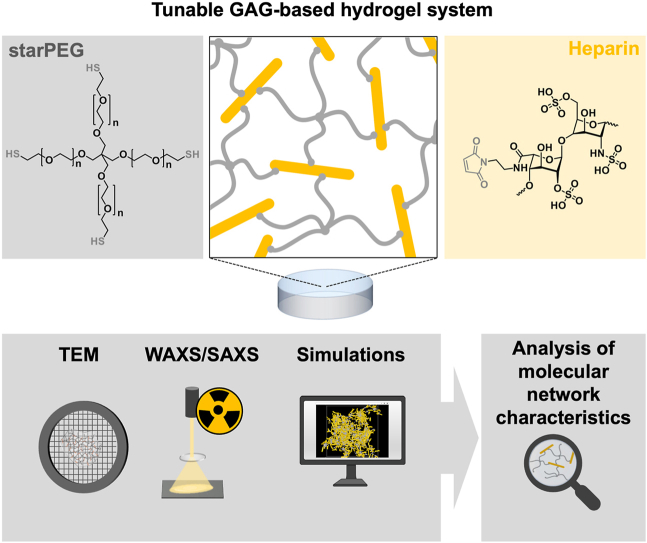

## 1 Introduction

Hydrogels – swollen hydrophilic polymer networks – can be precisely tuned to mimic the physical and biochemical properties of the native extracellular matrix (ECM) of soft tissues [[1], [2], [3]], making them particularly attractive for biomedical applications such as cell-instructive scaffolds or drug delivery vehicles [[2], [3], [4]]. The intrinsic structure and function these materials are largely determined by the nature of their polymer building blocks and the cross-linking strategies used to assemble their networks [5]. Key network properties, including mesh size, swelling degree and stiffness, are critical for controlling the diffusion of nutrients, oxygen, drugs, and signalling molecules, which in turn control cellular behavior like growth, migration, and differentiation [3,5].

Engineered hydrogels based on synthetic polymers – particularly poly(ethylene glycol) (PEG) – are chemically defined and allow for a far-reaching control over these network parameters [6,7]. However, the fundamental structural elements of these materials – the nanometer-sized polymer chains – are not directly observable using conventional imaging techniques such as confocal laser scanning microscopy or scanning electron microscopy. Although transmission electron microscopy (TEM) provides the necessary resolution, it typically demands contrasting methods based on charge interactions between the sample and staining agents, which can potentially alter the native network structure [5].

Consequently, a comprehensive structural analysis of these polymer networks requires a combination of complementary techniques, as each method provides a unique view into the material's architecture. For instance, key network parameters like mesh size are often inferred from bulk mechanical properties using theoretical frameworks like rubber elasticity theory [[8], [9], [10], [11]]. While valuable, these approaches provide bulk-averaged values and rely on simplified models that can overlook local heterogeneities and critical defect structures, such as elastically ineffective loops and dangling ends [[11], [12], [13]]. In parallel, techniques like X-ray scattering (SAXS/WAXS) offer a direct probe of structure, providing quantitative, ensemble-averaged information on features like polymer conformation and ordered domains. However, this information resides in reciprocal space and must be interpreted through modeling to yield specific real-space parameters. Complementing this, high-resolution imaging like TEM provides direct, real-space visualization of network morphology, but its reliance on sample processing, such as dehydration and staining, must be carefully considered. Given that each of these techniques offers unique strengths but also has inherent limitations, and that engineered hydrogel platforms are becoming increasingly complex [14,15], there is an urgent need for integrated methodologies that correlate findings across these different modalities to build a more complete and robust understanding of their nanoscale architecture.

Addressing this need, we present an approach to structurally characterize the molecular network of glycosaminoglycan (GAG)-PEG biohybrid cell-instructive hydrogels by combining: (1) **Direct TEM imaging** for high-resolution visualization of the nanometer-scale polymer chains; (2) **Small/wide-angle X-ray scattering (SAXS/WAXS)** to assess building block conformation and nano-ordered network inhomogeneities; and (3) **Computer simulations** to model network structure and connectivity. This methodology is further complemented by an in-depth characterization of the physical (bulk) properties using atomic force microscopy (AFM) nanoindentation and swelling studies. The integration of these techniques offers complementary insights, enabling a quantitative assessment of the hydrated molecular network across different physical states and resolutions.

The investigated hydrogel platform consists of a synthetic four-armed PEG (starPEG) and the natural GAG heparin, which are covalently crosslinked through a cytocompatible crosslinking reaction that allows for direct cell encapsulation [16,17]. The rational design of the starPEG-heparin hydrogel system – with adjustable stiffness, cell adhesiveness and cell-responsive degradation – permits independent tuning of both biomolecular and physical attributes [18,19]. Its proven ability to deliver signalling proteins, create morphogen gradients, and scavenge inflammatory chemokines and cytokines via reversible electrostatic interactions with the heparin building block [20] has been extensively investigated [[21], [22], [23]]. Additionally, the modular nature of the hydrogel has enabled its applications in directing stem cell fate and morphogenesis [[24], [25], [26]], studying disease mechanisms [[27], [28], [29]], and scavenging inflammatory proteins for chronic wound treatment [30,31]. While the cell-instructive potential of this hydrogel platform has been extensively documented, the primary goal of the present study is to elucidate the fundamental nanoscale architecture that governs this bioactivity.

By combining advanced imaging, scattering techniques, and computational modeling, the nanoscale structure, building block distribution and network defects of the biohybrid hydrogel system have been visualized, characterized, and correlated with the physical properties of the hydrogels. This integrated approach not only correlates these nanoscale features with the macroscopic physical properties of the materials but also provides critical insights that are essential for optimizing the cell-instructive properties of the hydrogels for a wide array of biomedical applications.

## 2 Experimental section

**Preparation of starPEG-heparin hydrogels.** The hydrogel networks were formed by a cell-compatible Michael-type addition reaction between thiol end-functionalized starPEG molecules and maleimide-functionalized heparin, as described elsewhere [16]. Briefly, heparin (Mw: 14,000 g mol^−1^, Merck Millipore) was functionalized with maleimide groups, achieving an average of six groups per heparin molecule. For hydrogel formation, the heparin-maleimide and starPEG (Mw: 10,000 g mol^−1^, JenKem Technology) precursors were each dissolved separately in Phosphate-Buffered Saline (PBS), using half of the total hydrogel volume for each solution. The final hydrogel mixture contained a heparin concentration of 1.5 mM. The crosslinking degree of the hydrogel network was controlled by varying the molar ratio of starPEG to heparin-maleimide (γ). To prepare cylindrical hydrogel discs (9 mm in diameter and 1 mm in height), glass coverslips (9 mm diameter) were first hydrophobized by treatment with Sigmacote® (Sigma-Aldrich Merck KGaA). Immediately after mixing the starPEG and heparin-maleimide precursor solutions, 67 μL of the solution was quickly dispensed onto one glass coverslip and overlaid with a second one. The hydrogels were left to fully polymerize for 15 min at room temperature under a humidified atmosphere, after which the coverslips were carefully removed. Prior to any subsequent analyses, all hydrogel samples were thoroughly washed in PBS – at least three times – followed by a final overnight washing step.

**Preparation of pure starPEG hydrogels as reference**. Pure starPEG hydrogels were synthesized using a protocol analogous to that employed for starPEG–heparin hydrogels. In this modified approach, four-armed, maleimide-end-functionalized starPEG molecules (Mw: 10,000 g mol^−1^; JenKem Technology) replaced the heparin–maleimide.

**Characterization of swelling degree.** The diameter of each cylindrical hydrogel disc was measured directly after polymerisation and again after reaching equilibrium swelling in PBS using a fluorescent scanner (FLA 5100, Fujifilm), with measurements quantified using Multi Gauge V2.2 software. The volume swelling degree was calculated as follows:

(1) $$volume\;swelling\;degree={\left(\frac{d}{d_{0}}\right)}^{3}$$

where *d* is the diameter of the swollen hydrogel and $d_{0}$ the initial diameter of the cast hydrogel.

To determine the swelling behavior in ethanol – mimicking the conditions during TEM imaging – a sequential ethanol dilution series was performed. Hydrogels were incubated for at least 30 min in each of the following solutions: 30 % and 50 % ethanol in PBS, 70 %, 80 %, and 90 % ethanol in ultrapure water, and finally 100 % ethanol. After imaging with the fluorescent scanner, the samples were then incubated in the reverse order of the ethanol series to assess the reversibility of the swelling. To ensure complete removal of ethanol, two additional washing steps with pure PBS (each for 24 h) were carried out at the end.

**Oscillatory rheological analysis.** Before measurements, cylindrical starPEG-heparin hydrogel discs were prepared as described and trimmed to a diameter of 8 mm using a biopsy punch (pfm medical AG). Oscillatory rheology tests were performed on PBS-swollen hydrogels using a rotational rheometer (ARES-LN2, TA Instruments, Germany) equipped with an 8 mm parallel plate geometry. The measurements were conducted over a shear frequency range of 1–100 rad s^−1^ at a constant strain amplitude of 2 %. The storage modulus (*G*′) of each sample was recorded with Rheology Advantage software (TA Instruments).

Based on the classical theory of rubber elasticity, the mesh size (ξ) of the polymer network was calculated from the storage modulus using the equation [3,5]:

(2) $$mesh\;size\;\left(\xi \right)={\left(\frac{G^{\prime }N_{A}}{RT}\right)}^{-\frac{1}{3}}$$

where $G^{\prime }$ is the storage modulus, N_A_ is Avogadro's number, R is the universal gas constant, and T is the absolute temperature.

**AFM-based nanoindentation measurements.** To assess the mechanical stiffness of the hydrogels in both PBS and ethanol, a NanoWizard II AFM (JPK Instruments) integrated with an inverted light microscope (Axio Observer D.1, Zeiss) was employed. Before measurements, a tipless triangular cantilever (PNP-TR-TL-Au, Nanoworld) was modified with a 10 μm silica bead (∅10 μm; Kisker Biotec GmbH) and calibrated using the equipartition theorem [32]. Prepared cylindrical hydrogels were placed in Petri dishes containing either PBS or ethanol. These dishes were pretreated with a 0.1 % polyethyleneimine solution (Sigma-Aldrich Merck KGaA, Germany) to ensure firm adhesion of the hydrogels to the bottom. Nanoindentation was performed at room temperature in PBS using a set point of 6 nN and an approach/retract velocity of 5 μm/s. For each sample, at least 70 measurements were taken at different locations. Force–distance curves from the measurements were analyzed using the AFM's proprietary data analysis software, and Young's moduli were derived based on the Hertz model [33]. The determined Young's moduli are related to the storage moduli through a Poisson's ratio of 0.5 [34].

**TEM imaging.** Prior to TEM imaging, the samples were washed five times (5 min each) in ultrapure water and then incubated on ice overnight in a 1 % uranyl acetate solution (diluted in ultrapure water) for contrast enhancement. The samples were subsequently rinsed again in ultrapure water (4 times, 10 min each). For dehydration, the samples were sequentially incubated in an ethanol/water dilution series—30 %, 50 %, and 70 % ethanol for 20 min each—followed by 90 %, 96 %, and three rounds in 100 % ethanol (30 min each). Infiltration with the embedding resin EMBed 812 into the samples was achieved by incubating them in progressively concentrated EMBed 812/100 % ethanol mixtures (1:2, 1:1, and 3:1; 1 h each). After a final overnight incubation in pure EMBed 812, the samples were transferred into molds containing EMBed 812 and polymerized for 24 h at 60 °C. Ultrathin sections (70 nm) were then obtained using a Leica UC6 ultramicrotome (Leica Microsystems) equipped with a diamond knife. The sections were mounted on grids, counterstained with uranyl acetate, and imaged at 80 kV using either a Morgagni 268D (Thermo Fisher Scientific) or a Jeol JEM-1400 Plus (Jeol GmbH).

**Structural analysis of TEM images.** Automated analysis of TEM images was carried out using a custom macro developed in ImageJ-Fiji (ImageJ, NIH) (Fig. S3). First, the TEM image files were pre-processed by applying contrast enhancement and a Gaussian filter to improve image quality, followed by binarization to clearly delineate features.

For estimating the structural length of individual heparin molecules, the binarized images were skeletonized, and the resulting skeleton was analyzed. Here, each branch – representing segments connecting endpoints or junctions – was measured to quantify the molecular lengths.

To determine the average size of the void structures within the hydrogel, the binarized images were further processed using a distance mapping algorithm and local maxima detection. A subsequent particle analysis provided measurements of the Feret diameter for these voids.

**X-ray scattering measurements.** Cylindrical hydrogels were sectioned into 400 μm slices at room temperature using a semi-automatic vibrating blade microtome (Vibratome VT1200, Leica Biosystems). The sections were then dehydrated via a graded ethanol series and finally dried in a heating oven at 30 °C (Vacutherm, Heraeus). SAXS/WAXS analysis was conducted using a laboratory X-ray diffraction system (NanoStar, Bruker AXS) with a wavelength of 0.154 nm. For each hydrogel, measurements were recorded from both the center and the border of the discs. Instrument-related background scattering was corrected, and the two-dimensional scattering data were integrated to yield the intensity (I) as a function of the scattering vector q.

**Computational modelling of starPEG-heparin hydrogel networks.** A detailed description of the methodology and the parameters used is provided in the Supporting Information. Briefly, the crosslinking, (de)swelling, and solvent interactions in starPEG–heparin hydrogels were modelled using Monte Carlo simulations within the Bond Fluctuation Model (BFM) framework [35,36]. In this coarse-grained approach, flexible polymers are represented as connected monomers (cubes) on a simple cubic lattice that undergo random Brownian motion in an implicit solvent. The Monte Carlo sampling was performed by random selection and random displacement of monomers obeying bond vector length constraints and lattice occupancy (excluded volume). Throughout all simulations, this ensured topology preservation and cut-avoidance. As a basic time unit, one Monte Carlo step (MCS) was defined as one attempted monomer move on average. The length of one lattice site was set to unity uBFM = 1, which can be mapped by the segment length of PEG 7.1 Å to be uBFM ≈3 Å [37]. Therefore, starPEG is modelled as a tetra-functional star polymer with 29 statistical monomers per arm, whereas heparin is constructed as a bulky, rod-like structure consisting of 90 cubes. Before the crosslinking process, solutions similar to the experimental setup of starPEG and heparin under different molar ratios γ have been prepared in a 256^3^ uBFM^3^ simulation box and equilibrated for at least 1 × 10^8^ MCS. For the crosslinking procedure, the four terminal monomers of starPEG molecules and 28 randomly chosen monomers on heparin have been labelled as reactive sites. Within the BFM algorithm, a permanent bond will be formed by a direct collision between reactive sites of starPEG and heparin. The crosslinking simulation proceeds as long as necessary to obtain an extent of reaction p = 0.90, defined as the number of established crosslinks divided by the number of all starPEG terminal groups. At least 20 different starting conformations were used for the crosslinking process providing an ensemble average of the observables. The biggest molecule cluster (BMC), which is defined as the biggest connected structure within the crosslinked network, was extracted for further simulations and placed in a 512^3^ uBFM^3^ simulation box. The deswelling process in ethanol was modelled as attractive interaction of −0.30 k_B_T between starPEG monomers of the BMC to promote the collapse [38]. At least 6.5 × 10^7^ MCS for one simulation run are performed to model the dehydration and fixation of the ethanol dilution series. A conformational snapshot has then been taken, resembling the resin-embedded network. The BFM algorithm, simulations, and data processing were performed by the C++ framework LeMonADE, which was developed by us and is openly accessible and available free of charge in Zenodo [39].

## 3 Results & discussions

**Fig. 1:**
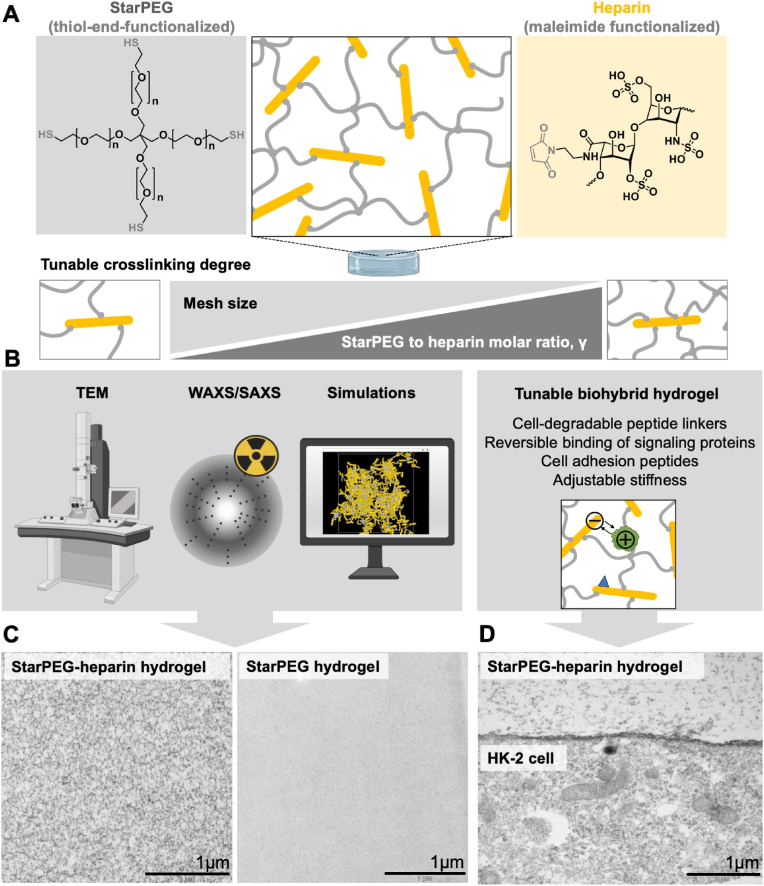
**Nanoscale structural analysis of starPEG-heparin hydrogels. A:** Schematic of the starPEG-heparin hydrogel, which is covalently crosslinked through a Michael-type addition between four-armed thiol-functionalized starPEG and maleimide-functionalized heparin. The crosslinking degree is controlled by varying the starPEG-to-heparin molar ratio (γ). **B:** Overview of the three complementary techniques used to analyze the nanometer-scale structure of the polymer networks. **C:** Representative TEM images of uranyl acetate-stained starPEG–heparin hydrogels and starPEG hydrogels (projection of ∼70 nm slices). Scale bars: 1 μm. **D:** TEM image (uranyl acetate staining) of human immortalized proximal tubule epithelial cells (HK-2) embedded in a cell-degradable starPEG-heparin hydrogel after four weeks of *in vitro* culture, highlighting the cell-hydrogel interface. Scale bar: 1 μm.

**Physical characterization and TEM-based visualization of starPEG-heparin hydrogel networks** were performed on samples prepared with varying molar ratios of starPEG to heparin (γ), resulting in polymer networks with distinct degrees of crosslinking (Fig. 1A). The hydrogels were synthesized via a Michael-type addition reaction between thiol-end-functionalized starPEG and maleimide-functionalized heparin, which served as a multifunctional crosslinker with its six maleimide groups [16]. The swelling degree of the hydrogel networks in PBS decreased with increasing γ (Fig. S1A), as the expansion forces driven by excluded volume and osmotic pressure became increasingly counteracted by the network's retraction forces arising from the higher number of elastically active polymer strands [18,19]. Mechanical characterization by AFM-based nanoindentation revealed that the elastic moduli of the PBS-swollen hydrogels ranged from 0.6 kPa to 17.5 kPa, depending on the molar ratio of starPEG to heparin (γ = 0.5, 0.75, 1.0 and 1.5) (Fig. S1C), and demonstrated that the crosslinking degree could be tuned independently of the heparin concentration (Fig. S1B). Additionally, oscillatory rheological measurements (Fig. S2A), analyzed using the general theory of rubber elasticity, provided estimates of the mesh sizes of the networks, which decreased from 31 nm to 8 nm with increasing γ (i.e. crosslinking degree) (Fig. S2B). However, these values only serve as approximations, as they do not fully reflect the complexity of the polymer network, which contains a multifunctional crosslinking building block (heparin) and exhibits local inhomogeneities (Fig. S2C). To gain deeper insight into the nanoscale architecture, TEM imaging was combined with SAXS/WAXS analysis and mathematical simulation, as schematically illustrated in Fig. 1B.

**Fig. 2:**
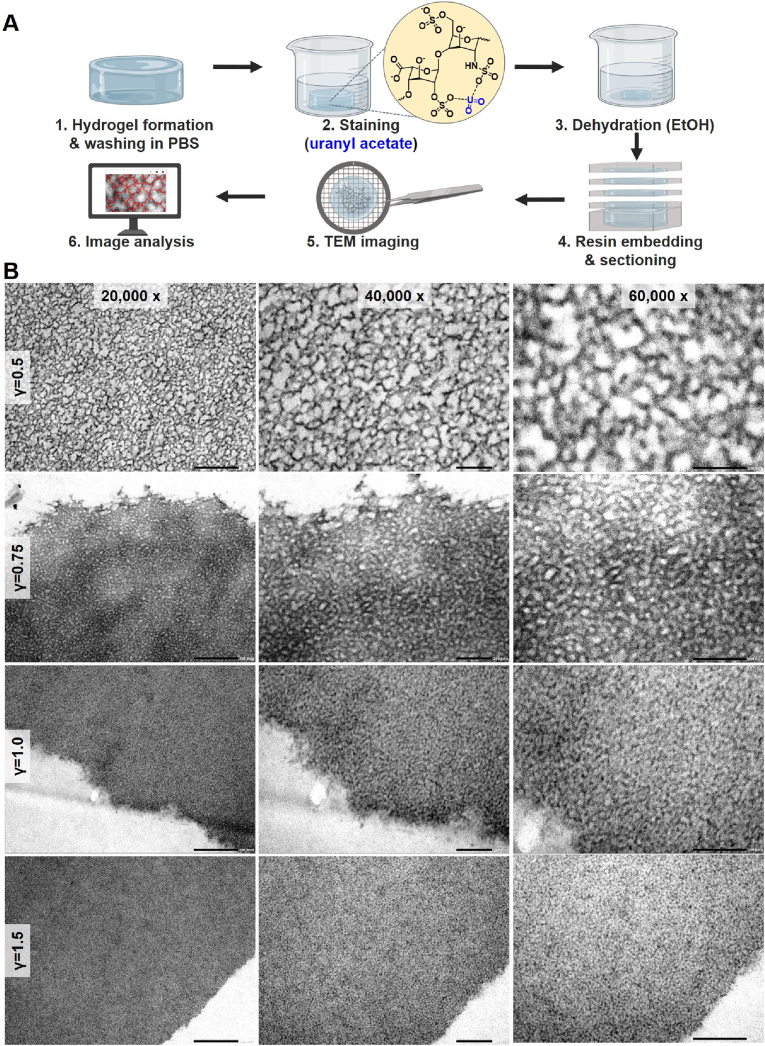
**TEM imaging of starPEG-heparin hydrogels. A:** Schematic of the TEM imaging workflow, including sample preparation steps involving ethanol (EtOH) dehydration and resin embedding. **B:** TEM images of starPEG-heparin hydrogels with varying starPEG-to-heparin molar ratio (γ) representing different crosslinking degrees. For each sample, 70 nm thin sections of ethanol-dehydrated, resin-embedded hydrogels are shown at different magnifications. All samples were stained with uranyl acetate (UA). The white regions (voids) correspond to starPEG-rich and resin-filled areas, whereas the dark regions represent UA-stained heparin. Scale bars: 500 nm for 20,000 × , 200 nm for 40,000 × and 60,000 × .

For TEM imaging, the starPEG-heparin hydrogels were first dehydrated in an ethanol series, then resin-embedded, sectioned and stained, as illustrated in Fig. 2A [5]. Consequently, the resin-embedded networks captured in TEM images (Fig. 2B) represent the equilibrium swelling state in ethanol. Under these conditions, the hydrophilic hydrogels exhibit significantly lower swelling compared to physiological conditions (i.e. in PBS) (Fig. S1A), due to both a reduction in counterion-induced osmotic pressure and the crystallization or aggregation of the polymeric components [40]. To confirm that the ethanol treatment does not cause permanent damage, we analyzed hydrogels re-swollen in PBS after the full dehydration procedure. A quantitative analysis showed that while the swelling degree exhibited some hysteresis, the elastic modulus was not statistically different from its pre-dehydration state (Fig. S1A and C). This preservation of mechanical properties, which are a direct measure of covalent network integrity, indicates that the core network structure is maintained through the dehydration-rehydration cycle, validating the preparation method for our structural analysis.

The TEM images clearly display the structural features of the starPEG–heparin networks (Fig. 2B). Importantly, UA staining selectively reveals the heparin building blocks of the network – appearing as dark structures – while the voids (white regions) correspond to the starPEG molecules and the surrounding resin present in polymer-free areas (mesh size). As expected, the size of the voids depends on the molar ratio γ of starPEG to heparin: hydrogels with lower crosslinking exhibit larger voids, indicative of a loosely connected network, whereas an increase in γ results in narrower voids, reflecting a more stable and homogeneous structure. High-magnification images from the network periphery (e.g., Fig. 2B, γ = 1 at 20,000 × ) further confirm that there is only a minimal background signal from the resin.

**Fig. 3:**
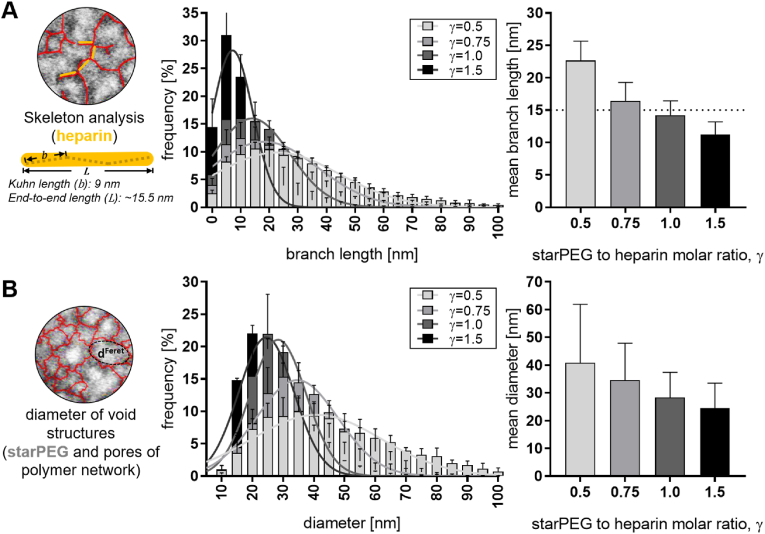
**Quantitative analysis of hydrogel nanoscale morphology using TEM image processing techniques. A:** Estimation of heparin molecule dimensions (rod-like structure) using ImageJ-Fiji's skeletonization algorithm. Results include the size distribution of branch lengths and the mean branch length derived from Gaussian fitting of the distributions. **B:** Quantification of void structure diameters (white regions) using ImageJ-Fiji's particle analysis tool. Frequency distributions of pore size and mean pore sizes are derived from Gaussian fitting of the size distributions.

**Characterization of network properties by TEM and X-ray scattering analysis.** The open-source software ImageJ-Fiji enabled extraction of detailed quantitative structural information from the TEM images. To determine both the structural length of the heparin molecules and to quantify the average size of the voids within the polymer network, two automated image processing methods were developed (Fig. S3). One method employed a skeletonization algorithm to extract quantitative branch lengths of the UA-stained heparin (visible as fine black lines in the TEM images; Fig. 3A, circle). The resulting size distributions indicated a mean branch length of approximately 15 nm (Fig. 3A) for intermediate crosslinking degrees (γ = 0.75 and 1), which aligns well with the previously reported 15.5 nm mean-square end-to-end distance for a similarly sized heparin molecule [41]. This confirms that the method effectively localizes the stiff heparin rods, corresponding to three Kuhn segments, within the nanometer-scale network.

Due to its high negative charge [20], the size of the heparin rods in ethanol is expected to remain consistent with measurements in PBS. In contrast, PEG, known to have lower hydrodynamic radii in alcoholic solutions compared to aqueous media [42], likely adopts an altered chain conformation in the dehydrated starPEG-heparin hydrogels. Analysis of the average branch length across hydrogels with varying crosslinking densities revealed a stepwise decrease from approximately 22 nm–12 nm as the molar ratio γ increased (Fig. 3A, right). This trend can be attributed to the higher polymer density in networks with increased γ, which leads to a greater incidence of overlapping heparin molecules within the 70 nm TEM sections and, consequently, fewer isolated heparin rods oriented perpendicular to the image plane (top view).

Furthermore, the skeleton analysis revealed that lower-crosslinked hydrogels (lower γ) exhibit a higher standard deviation and broader size distribution in branch lengths, reflecting higher structural heterogeneity. This observation is corroborated by the void structure analysis; as expected, the void diameters decrease with increasing γ, indicating denser network formation (Fig. 3B). Broader size distributions in lower-crosslinked hydrogels also suggest more heterogeneous defect structures in the softer networks (with Young's moduli ranging from 0.5 to 4 kPa), which is consistent with their significantly higher swelling response in PBS following network formation (Fig. S1 and A).

Since no single method can fully analyze a hydrogel network, integrating multiple analytical approaches provides a more complete understanding of its structure [5,43]. For example, TEM imaging coupled with X-ray diffraction has been used to examine self-assembled fibrillar networks in peptide-based hydrogels [43]. Following this method, SAXS/WAXS-analysis was performed on starPEG heparin hydrogels to complement TEM investigations. For X-ray scattering, the samples were dehydrated via an ethanol dilution series – without resin embedding – to minimize potential alterations from polymer packing that can occur during resin embedding. Recognizing that dehydration may induce structural changes, both dehydrated and fully hydrated (PBS-swollen) hydrogels were analyzed.

**Fig. 4:**
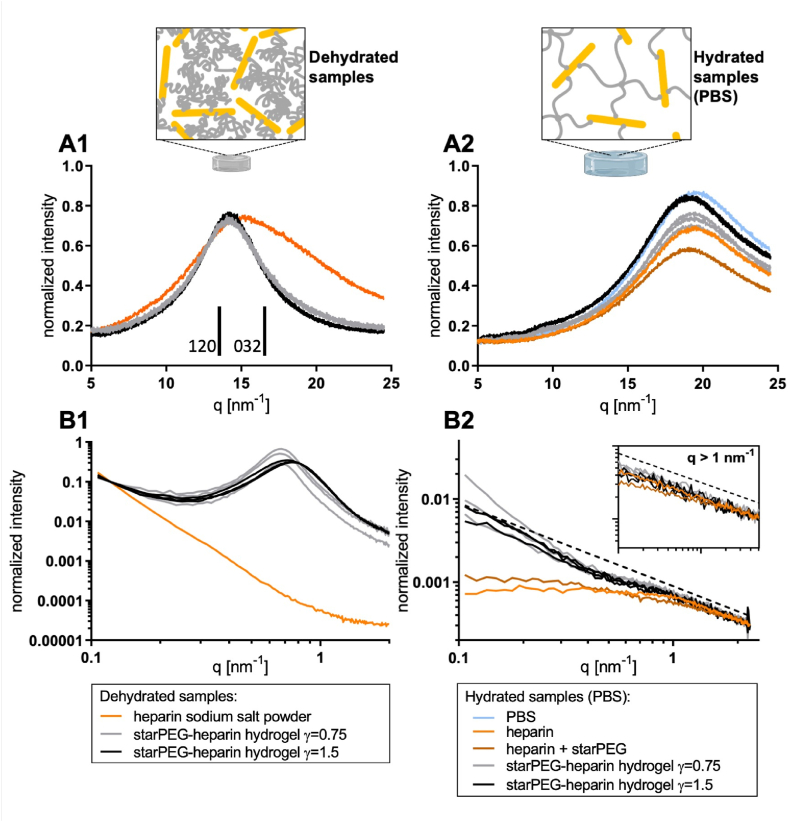
**X-ray diffraction analysis of starPEG-heparin hydrogels.** Diffraction patterns were recorded at three distinct measurement points within each hydrogel sample to assess potential network heterogeneities. The patterns were normalized to the average intensity at the lowest (A and B1) or highest (B2) q-values. **A:** WAXS diffraction patterns of dehydrated (A1) and equilibrium PBS-swollen (A2) starPEG-heparin hydrogels, as well as their precursors (heparin sodium salt and starPEG). A1: The positions of 120 and 032 Bragg reflections are indicated in the diffraction pattern. **B:** SAXS diffraction patterns of dehydrated (B1) and equilibrium PBS-swollen (B2) starPEG-heparin hydrogels and their precursors. The dotted line represents a curve with a slope of −1. Inset: Zoom of the q > 1 nm^−1^ region.

Fig. 4 presents the SAXS and WAXS intensity curves for starPEG–heparin hydrogels prepared at molar ratios (γ) of 0.75 and 1.5. For comparison, measurements were also performed on dry heparin sodium salt powder, heparin dissolved in PBS, and a PBS solution of a starPEG/heparin mixture, all at concentrations equivalent to those in the swollen hydrogels. In Fig. 4A1, the WAXS diffraction patterns for dehydrated hydrogels are shown alongside that of the heparin powder. The heparin powder exhibits a broad, amorphous peak typical of dehydrated polymers [44], whereas PEG polymers normally display distinct, narrow peaks in WAXS indicative of crystalline domains [[45], [46], [47]]. Notably, the hydrogel networks show a pronounced peak at q = 14.2 nm^−1^ (with a d-spacing of approximately 0.44 nm) that corresponds to the (120) crystal plane observed in crystalline PEG diffraction spectra [45,47,48]. However, the broad and symmetric nature of this peak is distinct from the characteristic asymmetric profile typically generated by the combined (120) and (032) reflections of semi-crystalline PEG. This suggests the signal does not necessarily arise from PEG crystallinity. A compelling alternative explanation is that the peak also reflects the local, nematic-like ordering of the rod-like heparin chains, an arrangement promoted by molecular confinement within the dehydrated network. Therefore, it is plausible that the observed signal is a convolution of both phenomena: short-range ordering of PEG chains and local anisotropic alignment of heparin.

This interpretation is further supported by the corresponding SAXS patterns (Fig. 4B1), where only the dehydrated hydrogel networks exhibit a distinct peak – absent in the amorphous heparin powder. These diffraction peaks represent a characteristic spacing of approximately 9.3 nm for γ = 0.75 and 8.5 nm for γ = 1.5. It is critical to note that these SAXS-derived distances are fundamentally different from the much larger, network-level mesh sizes observed by TEM (which decrease from ∼35 nm to ∼25 nm with increasing γ). Therefore, the SAXS peak in the dehydrated state does not represent the overall network mesh size. Instead, we propose that this signal originates from a more local, short-range ordering that emerges upon dehydration, likely reflecting the characteristic spacing between the semi-crystalline PEG domains that form when the solvent is removed.

In contrast, the hydrated hydrogels immersed in PBS exhibit only water scattering in the WAXS measurements (Fig. 4A2), and the distinct peaks observed in the dehydrated samples vanish completely from the SAXS profiles upon hydration [49]. This absence of defined peaks indicates that the ordered features in the dry state arise solely from the compact packing of the polymers, while in PBS the starPEG and heparin molecules are fully hydrated and any inherent order is lost.

Indeed, the SAXS patterns of the hydrated hydrogels are similar to those reported for uniformly hydrated networks [[50], [51], [52]]. PBS-dissolved heparin and the non-crosslinked starPEG/heparin mixture display comparable scattering profiles, suggesting that heparin governs the scattering behavior in solution (Fig. 4B2). At low q-values the SAXS patterns of hydrated starPEG-heparin hydrogels (Fig. 4B2) show increased scattering intensities compared to PBS-dissolved heparin or a non-crosslinked starPEG and heparin mixture, implying altered structural features resulting from the polymer network formation. In contrast, an almost identical scattering behaviour in the high q-regime (q > 1 nm^−1^) of hydrogel networks with different crosslinking degrees and the precursors in solution indicates the same molecular structure of heparin.

Overall, the scattering intensities of the starPEG-heparin hydrogel samples follow a slope of approximately −1 especially at q > 1 nm^−1^ (Fig. 4B2, dotted line), which is a characteristic signature of rod-shaped structures such as the heparin building blocks [41,53]. Estimations based on the thin rod limit of the form factor yield a length scale of about 3–4 nm, which is on the same order of magnitude as the reported Kuhn length of ∼9 nm for heparin [41].

Thus, the combined SAXS/WAXS data indicate that starPEG and heparin molecules remain fully hydrated in the PBS-swollen hydrogels, with no evidence of conformational changes of the bioactive heparin upon network formation. Preserving its rod-like architecture is critical for heparin's biomimetic interactions with soluble signaling molecules, as also observed in extracellular matrix and glycocalyx assemblies [54,55].

**Computational modelling of starPEG-heparin networks.** Based on TEM image evaluation and SAXS/WAXS data, computational simulations of starPEG-heparin hydrogels were performed by adapting a theoretical model previously used to describe the physical network properties of the hydrogel system [18,19]. Since X-ray analysis revealed the formation of semi-crystalline PEG-aggregates during dehydration, the simulations addressed both network formation in a good solvent (PBS) and the subsequent deswelling in a poor solvent (e.g., ethanol), with parameters set to enhance the self-aggregation of starPEG molecules. In contrast to heparin molecules that should not favor self-aggregation due to their high charge density, PEG-based hydrogels have been reported to form crystal-like aggregates in the dehydrated state [46,47], and PEG polymers were shown to form crystalline structures in ethanol [42].

In the simulation, starPEG is modelled as a four-armed star polymer with a tetrafunctional central monomer and reactive terminal groups. Heparin is represented as a rigid, rod-like multifunctional crosslinker providing up to 28 potential binding sites – based on the average number of carboxylic acid groups on a heparin molecule, which were covalently functionalized with stochastically distributed maleimide groups (Fig. S4) [16].

A defined number of starPEG (“nPEG”) and heparin (“nHEP”) molecules are introduced in the simulation setup to fix the molar ratio γ = nPEG/nHEP. For an ideal network structure, all heparin and starPEG molecules of the precursor solution should be quantitatively incorporated into the polymer network by forming single elastic bonds between one heparin and one starPEG molecule each. However, due to the statistical nature of the crosslinking reaction, imperfections (primitive defects) such as dangling starPEG arms or multiple bonds between one starPEG and a single heparin molecule occur (Fig. S5A). Moreover, the limited extent of reaction (p = 0.9 assumed) will lead to unbound heparin and starPEG in the reaction mixture, which do not contribute to the network properties but rather will be leached upon swelling. By removing these unbound components, the biggest molecule cluster (BMC) of the network was extracted, containing only crosslinked network components, characterized by an effective molar ratio γ_BMC_. BMCs from networks with different crosslinking densities were used for all further analyses (Table S2).

To model the network's deswelling in ethanol, an attractive interaction between starPEG molecules was introduced to promote self-aggregation, mimicking the formation of semi‐crystalline PEG domains observed from the scattering data. Throughout the simulation, both starPEG and heparin molecules undergo thermal motion within an implicit solvent. Further details on the computational modelling are provided in the Supporting Information.

**Fig. 5:**
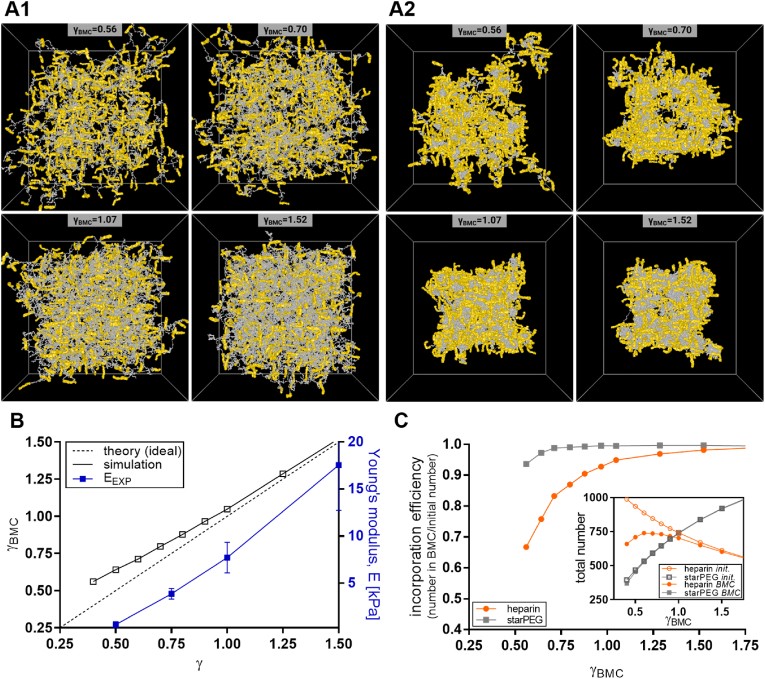
**Computational modelling of starPEG-heparin hydrogel networks. A:** Simulation snapshots of hydrated and dehydrated starPEG-heparin hydrogels. For the hydrated network (A1), a good solvent (equivalent to PBS) was assumed. To model the dehydrated state (A2), parameters were adjusted to promote the self-aggregation of starPEG molecules in a poor solvent (e.g., ethanol). Networks with different effective molar ratios after crosslinking (γBMC, where BMC is the Biggest Molecule Cluster) are shown, assuming a 90 % extent of reaction between starPEG (grey) and heparin (yellow). Cube size: L = 150 nm. **B:** Molar ratio of the BMC γ_BMC_ after crosslinking at an extent of reaction p = 0.9, plotted as a function of the initial molar ratio. The dotted line represents the theoretical ideal value, while the blue line shows the experimentally determined Young's moduli as a function of crosslinking degree. **C:** Incorporation efficiency of starPEG and heparin within the BMC, calculated as the number of starPEG or heparin molecules in the BMC divided by the number in the reaction mixture (ideal network). *Insert:* Total number of starPEG or heparin molecules in the reaction mixture (initial) or within the BMC. (For interpretation of the references to colour in this figure legend, the reader is referred to the Web version of this article.)

Fig. 5 shows snapshots of the simulated starPEG-heparin hydrogel networks in both good (A1) and poor (A2) solvent. The images reveal that, with increasing starPEG/heparin molar ratios (γ), the networks become progressively denser and more compact. This densification correlates with higher Young's moduli, as confirmed by AFM nanoindentation measurements on PBS-swollen networks (Fig. 5B, blue line). In the deswollen (ethanol treated, resin-embedded mimic) simulations, networks with the lowest γ value (γ = 0.5) are characterized by large voids – indicative of extensive polymer-free regions – and starPEG aggregates crosslinked by heparin that form loosely bound “strut-like” structures. These features correlate with the larger voids observed in TEM images at lower γ values and may explain why the UA-stained heparin regions appear “thicker” under these conditions (Fig. 2B).

Conversely, as γ increases, the enhanced incorporation of starPEG leads to a more homogeneous heparin distribution and overall denser, more stable networks (Fig. 5B). Moreover, based on the 3D models of dehydrated starPEG-heparin hydrogels, it can be assumed that for low γ-values the white regions on the TEM images are a combination of resin (polymer-free space) and starPEG self-aggregates.

The results can also be interpreted through a quantitative evaluation of the BMC. In Fig. 5B, the effective γ_BMC_ of crosslinked starPEG-heparin hydrogels and the experimentially determined Young's moduli are plotted against the reaction mixture's γ. At lower γ values, γ_BMC_ deviates strongly from the theoretical γ, indicating a significantly lower incorporation of building blocks and, thus, a higher number of severe defects (Fig. 5B). In particular, heparin incorporation is markedly lower (see incorporation efficiency, Fig. 5C), which is attributable to the low theoretical number of elastically active starPEG–heparin bonds. For example, with f_HEP,BMC_ = 2.03 for γ_BMC_ = 0.56 (corresponding to a reaction mixture of γ = 0.4), the functionality lies just at the threshold necessary for successful network formation, since a minimum functionality f_HEP_ > 2 is required given the 4-arm structure of starPEG as a second building block. Consequently, even a few defects significantly impair network connectivity, leading to substantial fluctuations and an increased number of dangling heparin molecules, thereby enhancing network heterogeneity.

As the initial molar ratio γ increases, the heparin functionality *f*_HEP,BMC_ increases (Table S2), meaning that on average more starPEG molecules are bound per heparin molecule. This increased functionality permits more imperfections while still maintaining sufficient connectivity, resulting in a higher number of elastically active strands and, consequently, denser and more stable networks. This behavior aligns with the experimentally determined Young's moduli (Fig. 5B) and the increased cycle rank per strand ζ of the network (Fig. S5B), a fundamental invariant of network connectivity related to the elasticity as the prefactor in the phantom network model [9].

Overall, the network inhomogeneities observed in the TEM images (Fig. 2) and in the simulation snapshots (Fig. 5A) at low γ-values can be explained by a combination of lower heparin incorporation efficiency, higher network fluctuations with resulting dangling heparin molecules loosely bound to the BMC, as well as more-pronounced starPEG-aggregate formation. In contrast, at higher γ-values, smaller PEG aggregates and a more compact network are observed, as evidenced by denser structures in the TEM images (Fig. 2B). Thus, the simulation of the starPEG–heparin hydrogel not only complements the structural insights provided by TEM and SAXS/WAXS measurements but also suggests that the void size in TEM images can serve as a practical measure of "network quality" with large voids pointing to large defect structures.

## 4 Conclusions

In this study, we establish an integrated, multi-scale methodology to quantitatively characterize the nanoscale architecture of starPEG-heparin biohybrid hydrogels. By combining direct-space imaging (TEM), reciprocal-space analysis (X-ray scattering), mechanical testing (AFM), and computational modeling, our approach successfully links molecular-level design to macroscopic functional properties.

A central achievement is the direct visualization of the bioactive heparin component, which is essential for the material's ability to sequester and present signaling molecules to cells. Our TEM analysis reveals a mean branch length for heparin of approximately 15 nm, a value in excellent agreement with the reported 15.5 nm mean-square end-to-end distance for a heparin building block of similar molecular weight [41]. Critically, this is complemented by SAXS data confirming that heparin retains its functionally essential, rod-like conformation [20,53] upon network incorporation. This provides direct structural evidence for the preservation of its bioactivity, confirming the structural basis for the biomimetic function that underpins this material's proven cell-instructive capacity [17].

Our approach represents a significant advance over traditional methods for hydrogel characterization. Typically, network properties like mesh size are indirectly estimated from bulk mechanical measurements using theories of rubber elasticity [[8], [9], [10], [11]]. Such methods provide only averaged values and cannot account for the local heterogeneities and network defects that critically influence material performance [12,13]. Our work moves beyond these limitations. By directly imaging the network, we visualized structural inhomogeneities and correlated them with crosslinking density. We demonstrated that higher crosslinking degrees (γ) yield more homogeneous structures with smaller voids and fewer defects, which directly corresponds to their increased mechanical stiffness and controlled swelling behavior. This provides a tangible, visual link between nanoscale architecture and bulk properties, a goal that is central to the rational design of biomaterials.

Furthermore, our computational model provides the mechanistic underpinnings for these observations. The simulations reveal that inefficient heparin incorporation at low crosslinking densities leads to a higher prevalence of network defects, which in turn drives the formation of larger aggregates and voids upon dehydration. This synergy between simulation and experiment validates using TEM-derived void size as a practical, semi-quantitative measure of network quality and defect density.

In summary, this work unravels the structural basis of a widely used cell-instructive hydrogel system. By providing quantitative insights into network connectivity, building block conformation, and defect formation, our integrated methodology offers a robust framework for the rational design and quality control of advanced biomaterials. The ability to visualize the cell-hydrogel interface at the nanoscale (Fig. 1D) further opens exciting avenues to study dynamic processes like cell-mediated matrix remodeling, which is critical for applications in regenerative medicine, disease modeling, and tissue engineering.

## CRediT authorship contribution statement

**Jana Sievers-Liebschner:** Writing – original draft, Investigation, Formal analysis, Data curation. **Ron Dockhorn:** Writing – original draft, Investigation, Formal analysis, Data curation. **Jens Friedrichs:** Writing – review & editing, Investigation, Formal analysis, Data curation. **Thomas Kurth:** Investigation. **Peter Fratzl:** Writing – original draft, Investigation, Formal analysis. **Jens-Uwe Sommer:** Writing – review & editing, Supervision, Investigation. **Carsten Werner:** Writing – review & editing, Supervision, Funding acquisition. **Uwe Freudenberg:** Writing – review & editing, Supervision, Funding acquisition, Conceptualization.

## Declaration of competing interest

The authors declare that they have no known competing financial interests or personal relationships that could have appeared to influence the work reported in this paper.

## Data Availability

Data will be made available on request.
