# Supplementary material for: Unravelling the molecular network structure of biohybrid hydrogels
Source: Mater Today Bio. 2025 Aug 26;34:102249. doi: 10.1016/j.mtbio.2025.102249 (PMC12415082; doi:10.1016/j.mtbio.2025.102249)
Supplement: Multimedia component 1 [file mmc1.docx]

**Supporting Information: Unravelling the molecular network structure of cell-instructive hydrogels**

Jana Sievers-Liebschner^a^, Ron Dockhorn^c^, Jens Friedrichs^a^, Thomas Kurth^e^, Peter Fratzl^f^, Jens-Uwe Sommer^c,d^, Carsten Werner^a,b*^, Uwe Freudenberg^a*^

^a^Leibniz-Institut für Polymerforschung Dresden, Division Polymer Biomaterials Science, Max Bergmann Center of Biomaterials Dresden, 01069 Dresden, Germany

^b^Technische Universität Dresden, Center of Regenerative Therapies Dresden And Cluster of Excellence Physics of Life, 01069 Dresden, Germany

^c^Leibniz-Institut für Polymerforschung Dresden, Division Theory of Polymers, Dresden, 01069 Dresden, Germany

^d^Technische Universität Dresden, Institute for Theoretical Physics, And Cluster of Excellence Physics of Life, 01069 Dresden, Germany

^e^Technische Universität Dresden, Center for Molecular and Cellular Bioengineering (CMCB), Technology Platform Core Facility Electron Microscopy and Histology, 01307 Dresden, Germany

^f^Max Planck Institute of Colloids and Interfaces, Department of Biomaterials, 14476 Potsdam, Germany

*Corresponding authors: werner@ipfdd.de, freudenberg@ipfdd.de

I. SUPPLEMENTARY RESULTS: PHYSICAL CHARACTERIZATION OF STARPEG-HEPARIN NETWORKS

| 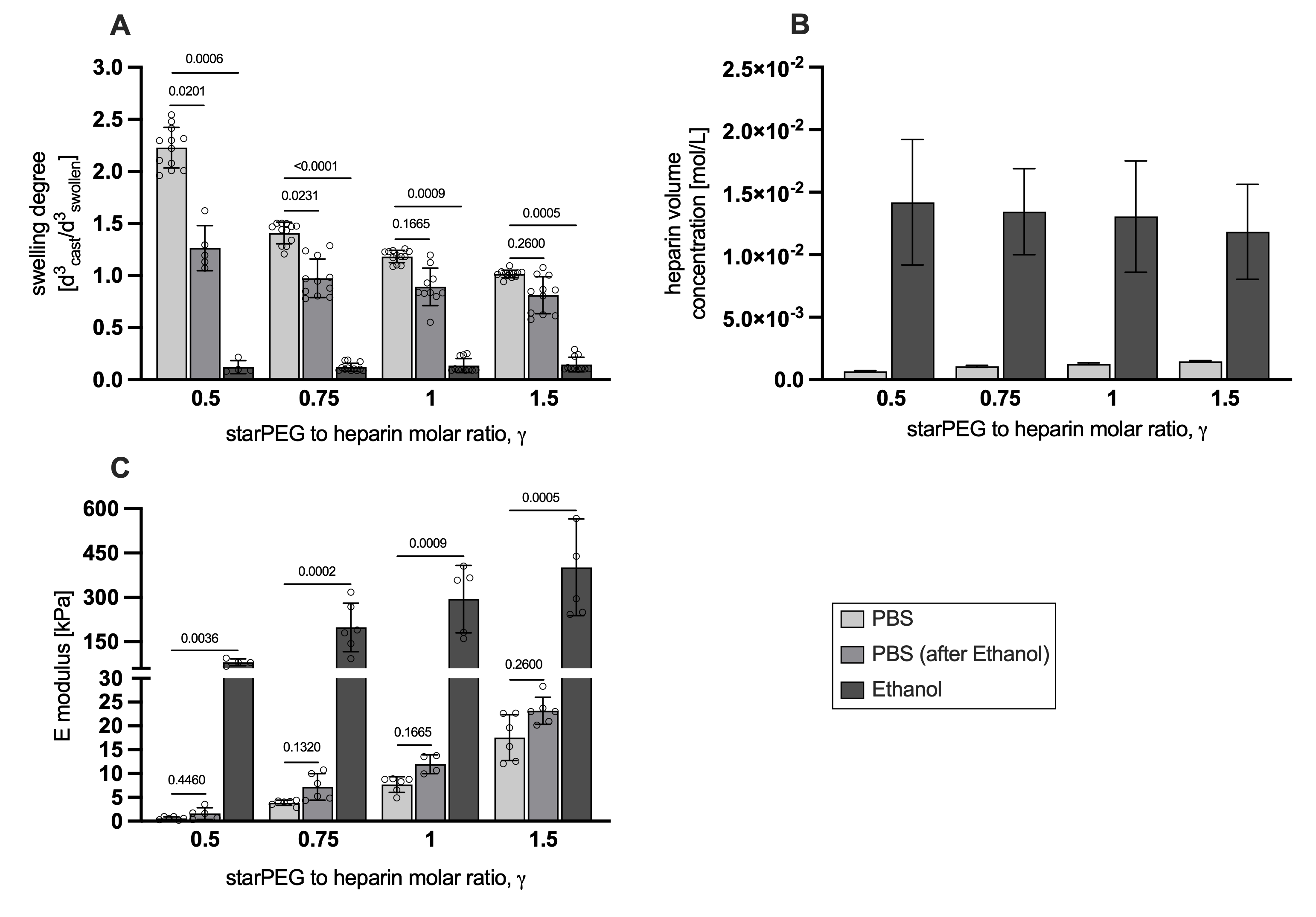 |
| --- |
| Figure S1. Swelling Behavior and Mechanical Properties of starPEG-Heparin Hydrogels. All analyses were performed on hydrogels swollen in PBS (physiological conditions), swollen in ethanol (conditions during TEM imaging), and re-swollen in PBS after ethanol dehydration (to assess swelling reversibility). (A) Quantification of equilibrium volumetric swelling degree. (B) Heparin volume concentration in PBS and ethanol, calculated from experimentally determined swelling degrees and the known heparin content used during hydrogel fabrication. (C) Mechanical properties assessed by AFM-based nanoindentation. Statistical comparisons were performed using a Kruskal-Wallis test; p-values are indicated. |

| 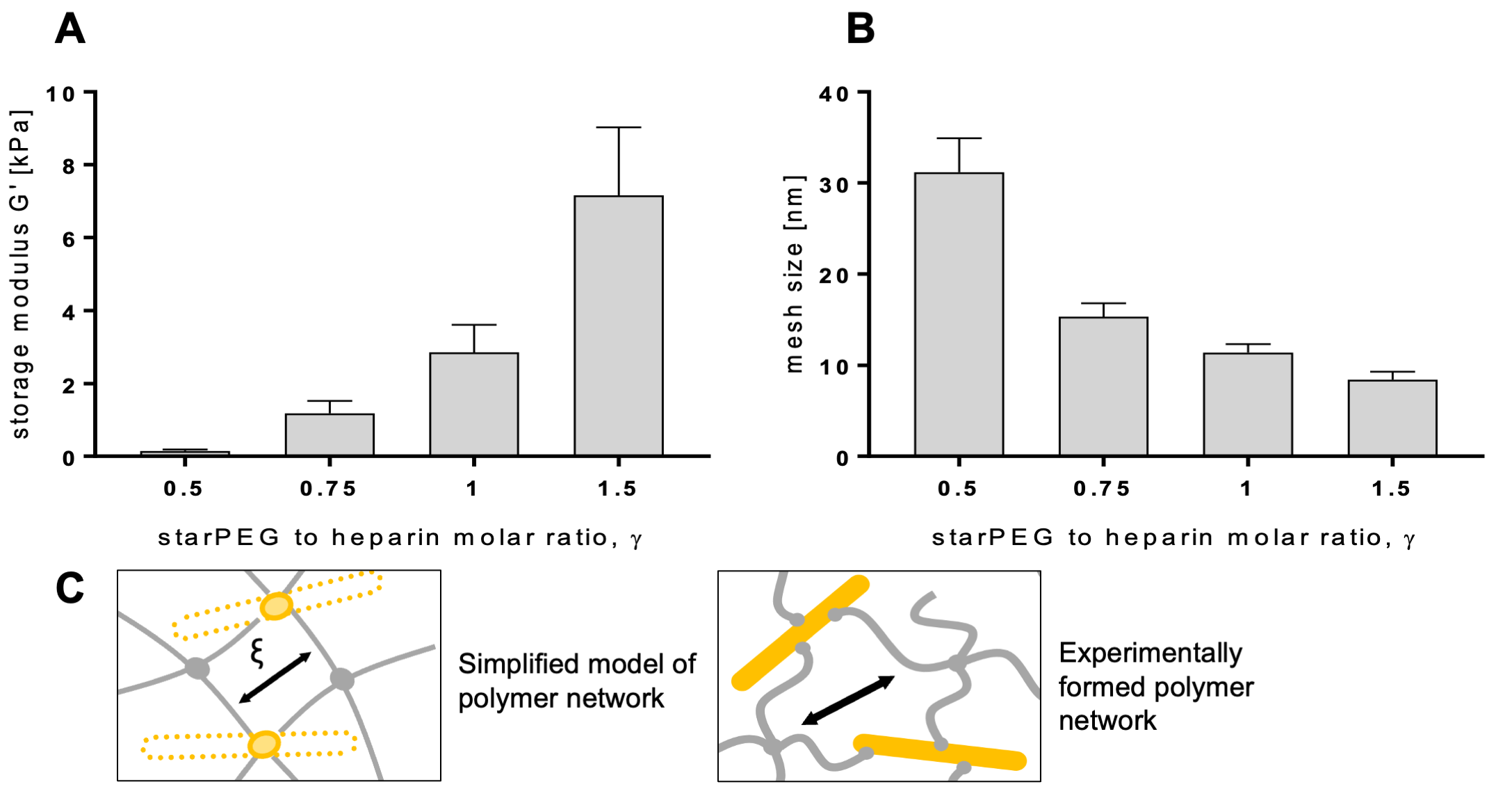 |
| --- |
| Figure S2. Rheological Characterization and Mesh Size Estimation of PBS-Swollen starPEG-Heparin Hydrogels. (A) Storage moduli of starPEG-heparin hydrogels determined by oscillatory rheology. (B) Hydrogel network mesh sizes calculated from rheological data using the general theory of rubber elasticity. (C) Schematic illustration of starPEG-heparin polymer network mesh size: (Left) Simplified theoretical model based on rubber elasticity theory; (Right) Experimentally formed heterogeneous network illustrating structural defects and heparin as a multifunctional crosslinker. |

II. SUPPLEMENTARY METHODS: STRUCTURAL ANALYSIS OF TEM IMAGES

| 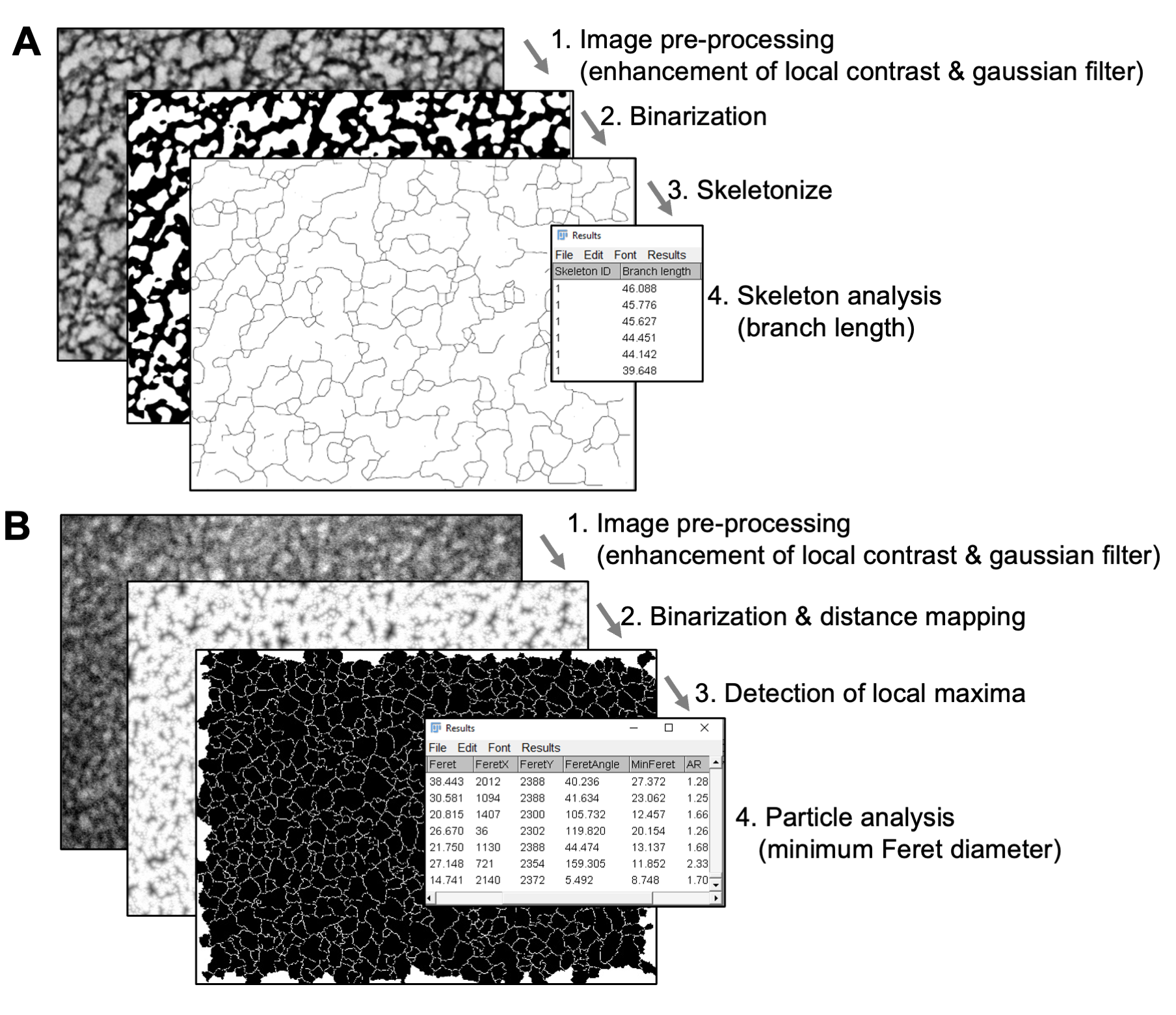 |
| --- |
| Figure S3. Structural Analysis of TEM Images Using ImageJ-Fiji Software. Step-by-step workflow illustrating the structural analysis of TEM images performed with the open-source image analysis software ImageJ-Fiji. (A) Estimation of structural length of heparin molecules. (B) Quantification of average void structure sizes. |

III. SUPPLEMENTARY METHODS AND RESULTS: COMPUTATIONAL MODELING OF STARPEG-HEPARIN NETWORKS

1. **Simulation Method**

A Monte Carlo approach is used in the framework of the Bond Fluctuation Model (BFM)[1,2] to model the crosslinking and swelling process of the starPEG-heparin networks in three dimensions. Flexible polymeric structures are represented by connected coarse-grained effective monomers(cubes) occupying eight edges on a simple cubic lattice, where the length of the unit cell is set to unity u_BFM_ = 1. The lattice serves as an efficient look-up table for the short-range monomer-monomer interaction. The excluded volume interaction is respected by prohibiting multiple occupations of a single edge by several monomers. The connectivity between monomers is established by 108 bonds out of a set of bond vectors $\vec{B}$ with six base vectors given by[2]

$$\vec{B}=P_{\pm}\left( \begin{aligned} 2 \\ 0 \\ 0 \end{aligned} \right) \cup P_{\pm}\left( \begin{aligned} 2 \\ 1 \\ 0 \end{aligned} \right) \cup P_{\pm}\left( \begin{aligned} 2 \\ 1 \\ 1 \end{aligned} \right) \cup P_{\pm}\left( \begin{aligned} 3 \\ 0 \\ 0 \end{aligned} \right)\cup P_{\pm}\left( \begin{aligned} 3 \\ 1 \\ 0 \end{aligned} \right), (1)$$

where P_±_ denotes all permutations and sign combinations of a triple. A trial monomer move in the framework of the Monte Carlo procedure works as follows: a monomer is randomly chosen, and a displacement move along a randomly chosen direction along one out of six unit cell lattice vector will be tried. Additionally, a Metropolis criterion is applied if the move results in an energy change of the system incorporating thermal interactions. The move will be performed if all constraints are satisfied; otherwise rejected. The algorithm is repeated as long as necessary to equilibrate the system and to sample the observables. As a basic time unit, one Monte Carlo step (MCS) is defined as one attempted monomer move on average. The BFM reproduces Rouse-like dynamics with implicit solvent, where no hydrodynamic interactions are present. The excluded-volume condition as well as the bond vector set ensures cut-avoidance with local and global topology conservation, where entanglements are considered. The BFM algorithm, simulations, and data processing are performed by the C++ framework LeMonADE developed in our group and is openly accessible and available free of charge in Zenodo [3].

StarPEG and heparin have been modeled in the framework of the BFM to investigate the network structure, the crosslinking procedure, and the solvent interactions. Here, starPEG is modeled as a 4-arm flexible star polymer with a tetrafunctional core monomer, whereas heparin serves as a bulky rod-like multi-functional crosslinker, see Fig. S4.

The number of statistical monomers representing starPEG *N*_PEG_ within the BFM is mapped according to the monomeric average length *b*_PEG_ = 2.78 Å in water[4] and the Kuhn segment length of about $L_{K}^{PEG}$ = 7.1 Å [4]. One star arm with molar mass $M_{PEG}^{arm}$ = 2500g/mol corresponds to 57 chemical monomers, which can be mapped to $N_{PEG}^{A}$ ≈ 1.3 · $\frac{2.78*57}{7.1}$ ≈ 29coarse-grained monomers within the BFM taking into account an additional factor [5] for the statistical segment length. Thus, starPEG is represented with a total of *N*_PEG_ = 117 monomers within the BFM. Furthermore, the length of the unit cell corresponds to be uBFM ≈ 3 Å.

| 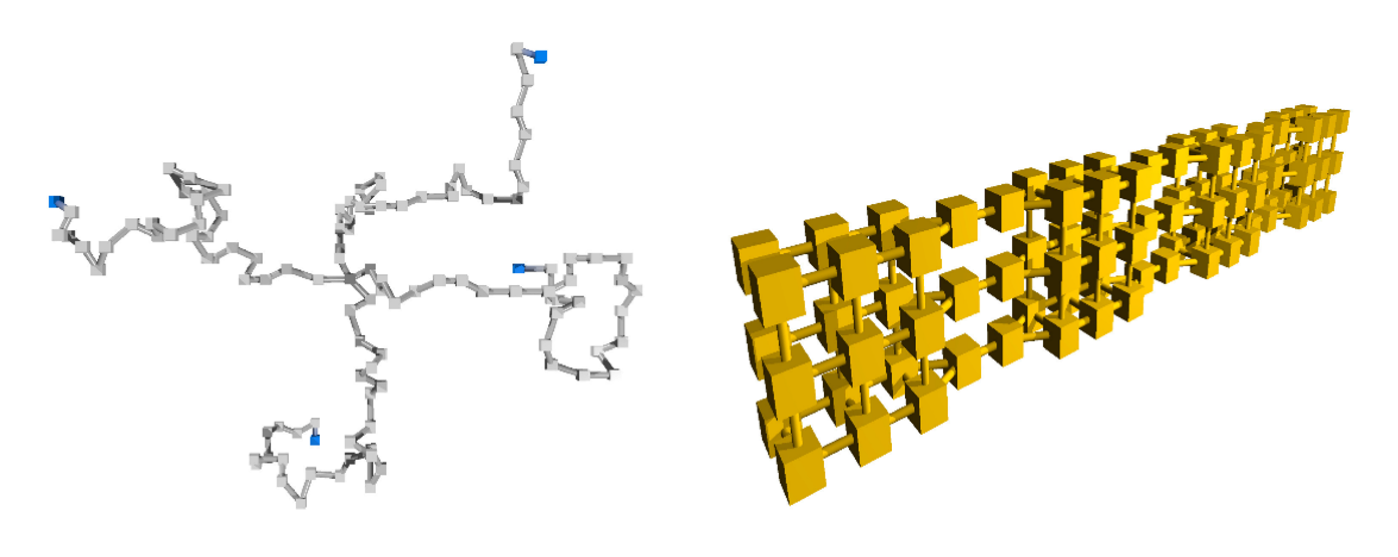 |
| --- |
| Figure S4. Scheme of different models for representing starPEG and heparin in the bond fluctuation model. Left: starPEG is modeled as a 4-arm star polymer with *N*_PEG_ = 117 monomers with a tetrafunctional center monomer and reactive monomers at the terminal groups (blue). Right: heparin is modeled as a highly connected structure with *N*_HEP_ = 90 monomers formed by space-filling blocks connected by bridging monomers for enhanced flexibility. |

In contrast, heparin, as a highly multi-functional crosslinker, is modeled as a rod-like structure to match the high Kuhn segment length $L_{K}^{PEG}$ ≃ 9nm [6]. As the BFM is designed only for flexible polymeric structures, the additional stiffness is introduced by four bulky, highly connected blocks resembling the experimentally obtained contour length *L*_HEP_ = 25nm [6]. Considering the molar volume ratio between starPEG and heparin δ = 0.885 [7], a value of *N*_HEP_ = 90 statistical monomers within the BFM is chosen to represent heparin as a bulky rod-like filler molecule providing enough attachment points for the crosslinking procedure.

As for the experimental setup *n*_PEG_ starPEG and *n*_HEP_ heparin molecules with the initial molar ratio $\gamma= \frac{n_{PEG}}{n_{HEP}}$ are placed in a 256^3^ $u_{BMF}^{3}$ cubic simulation box before crosslinking. The experimental overlap concentration $c_{PEG,exp}^{*}$ =6 · 10^−3^ μmol · μl^−1^ [8] can be mapped to the overlap concentration of starPEG in the BFM to be $c_{PEG}^{*}$ ≃ 0.044 allowing the estimation of the starPEG concentration in the simulation and to calculate the number of molecules, respectively. An overview of all simulation parameters for the preparation condition can be found in Tab. S1.

| \| *γ* \| *n*_PEG_ \| *n*_HEP_ \| *c* \| *f*_HEP,ideal_ \| \| --- \| --- \| --- \| --- \| --- \| \| 0.4 \| 395 \| 988 \| 0.064 \| 1.6 \| \| 0.5 \| 468 \| 936 \| 0.066 \| 2.0 \| \| 0.6 \| 533 \| 888 \| 0.068 \| 2.4 \| \| 0.7 \| 593 \| 847 \| 0.069 \| 2.8 \| \| 0.8 \| 646 \| 808 \| 0.071 \| 3.2 \| \| 0.9 \| 696 \| 773 \| 0.072 \| 3.6 \| \| 1.0 \| 741 \| 741 \| 0.073 \| 4 \| \| 1.25 \| 839 \| 671 \| 0.076 \| 5 \| \| 1.5 \| 920 \| 613 \| 0.078 \| 6 \| | Table S1. Preparation conditions and overall concentration *c* for cross-linking the coarse-grained *n*_HEP_ heparin and *n*_PEG_ starPEG molecules in the BFM to form the heparin-starPEG nanogels in a simulation volume 256^3^ $u_{BMF}^{3}$. To form an ideal network *f*_HEP,ideal_ = 4 · *γ* functional groups on heparin are necessary at a specific molarratio *γ*. |
| --- | --- | --- | --- | --- | --- | --- | --- | --- | --- | --- | --- | --- | --- | --- | --- | --- | --- | --- | --- | --- | --- | --- | --- | --- | --- | --- | --- | --- | --- | --- | --- | --- | --- | --- | --- | --- | --- | --- | --- | --- | --- | --- | --- | --- | --- | --- | --- | --- | --- | --- | --- |

The preparation of the solutions and the crosslinking procedures are performed under non-periodic boundary conditions with hard walls. The starPEG-heparin solutions in the BFM are equilibrated for at least 100 · 10^6^ MCS. After equilibration, conformations are extracted for every 2 · 10^6^ MCS,and at least 20 separate simulation runs are performed for the network formation process. For the crosslinking procedure, the four terminal groups on starPEG are considered ideal reactive, implying equal turnover rates during the crosslinking. For heparin, seven reactive groups per block are equally distributed on the rod-like structure corresponding to the average number of carboxylic acid groups per molecule, which have been functionalized with maleimide groups in the experiment. The crosslinking step of starPEG-heparin is performed during the Monte Carlo step within the displacement move: If two reactive heparin/starPEG monomers collide, an additional permanent bond is formed, and the monomers lose their reactivity. The number of additional crosslinks *n*_HEP-PEG_ between heparin and starPEG ranges between [0; 4 · *n*_PEG_]. The extent of reaction *p* can then be defined as

$$p= \frac{n_{HEP-PEG}}{{4n}_{PEG}}, (2)$$

reflecting the progress between no reaction (*p* = 0) to the fully saturated reaction of all stars (*p* = 1). The simulations ran as long as necessary to overcome the gelation point and stopped at *p* = 0.90 for comparison to the experiment. The conformations of the crosslinked nanogels are used for further analysis. For the simulation snapshots, one representative conformation at *p* = 0.9 at a particular molar ratio γ with the biggest molecule cluster (*γ*_BMC_, see below) is extracted and placed in a 512^3^ $u_{BMF}^{3}$ simulation box used for further simulations. Note that no electrostatic interaction between heparin is considered in the simulation, as the computational model represents the case in which excluded volume forces are dominating (both within the ethanol-resin preparation and the good solvent/high salt state). The swelling without any electrostatic interactions is purely driven by excluded volume interactions and corresponds to charge screening effects in high surrounding salt concentration in the experiment. Figure 5A1 in the main article shows the good solvent case for heparin (orange) and starPEG (gray) after 2 · 10^8^ MCS.

The deswelling process for starPEG in ethanol is modeled by an interaction shell between the nearest next neighbors according to [9]. An attractive energy per starPEG-starPEG contact ε_PEG-PEG_ = −0.30 is used, whereas no energetic interaction between other species ε_HEP-PEG_ = ε_HEP-HEP_ = 0.0 is applied. This energy scale promotes the collapse of polymer chains [10] and should lead to the self-aggregation of starPEG. The simulation runs with interactions were performed for at least 6.5·10^7^ MCS, and representative snapshots are taken for Figure 5A2 in the main article.#

1. **Biggest Molecule Cluster and Cycle Rank**

In contrast to the “ideal perfect network structure”, which only consists of elastically active single strands, primitive defects (Figure S5A, upper panel) evolve during the random cross-linking process, such as non-reacted starPEG arms that are not participating in the network structure (dangling ends),as well as multiple starPEG arms (double, triple, quadruple bonds) sharing the same heparin. Also, the limited extent of the reaction (*p* = 0.9) will lead to unbound heparin and dangling starPEG in the reaction mixture, which do not contribute to the network properties. Therefore, the biggest molecule cluster (BMC) of all heparin molecules *n*_HEP,c_ connected with all starPEG molecules *n*PEG,c forming the network is considered (Table S2). The molar ratio of the BMC is then defined by

$$\gamma_{BMC}\left( p \right)= \frac{n_{PEG,c}\left( p \right)}{n_{HEP,c}\left( p \right)}. (3)$$

| \| *γ* \| *γ_BMC_* \| *f*_HEP,BMC_ \| *f*_HEP,0.9_ \| \| --- \| --- \| --- \| --- \| \| 0.4 \| 0.56 \| 2.03 \| 1.44 \| \| 0.5 \| 0.64 \| 2.32 \| 1.80 \| \| 0.6 \| 0.71 \| 2.57 \| 2.16 \| \| 0.7 \| 0.80 \| 2.88 \| 2.52 \| \| 0.8 \| 0.88 \| 3.17 \| 2.88 \| \| 0.9 \| 0.97 \| 3.49 \| 3.24 \| \| 1.0 \| 1.05 \| 3.79 \| 3.60 \| \| 1.25 \| 1.29 \| 4.64 \| 4.50 \| \| 1.5 \| 1.52 \| 5.50 \| 5.40 \| | Table S2. The molar ratio of starPEG-heparin hydrogel networks in the initial *γ* and crosslinked *γ*_BMC_ (*p* = 0.9) state and the corresponding heparin functionality *f*_HEP,BMC_ of the crosslinked network in the BMC. For comparison, the expected heparin functionality *f*_HEP,0.9_ = 4*γ* · *p* at extent of reaction of *p* = 0.9 is also shown. |
| --- | --- | --- | --- | --- | --- | --- | --- | --- | --- | --- | --- | --- | --- | --- | --- | --- | --- | --- | --- | --- | --- | --- | --- | --- | --- | --- | --- | --- | --- | --- | --- | --- | --- | --- | --- | --- | --- | --- | --- | --- | --- |

Figure S5A shows the number of defects per heparin inside the BMC as function of the molar ratio *γ*_BMC_ at *p* = 0.9.For very low molar ratios the impact of the defects results in strong deviations from the ideal behavior. The limited heparin functionality, as well as the starPEG defects lead to networks with enhanced heparin content as a minority of heparin is not yet bound to structure, see Tab. S2. The simulations clearly show that when the molar ratio between both reactants increases, the total number of primitive starPEG defects per heparin molecule is increasing, as well. Hence, for lower crosslinked network (smaller γ-values) the total number of defects have a greater impact on the network quality, as their contribution is more significant to the average value of crosslinked PEG-arms per heparin molecule (Figure S5B,dashed line).

| 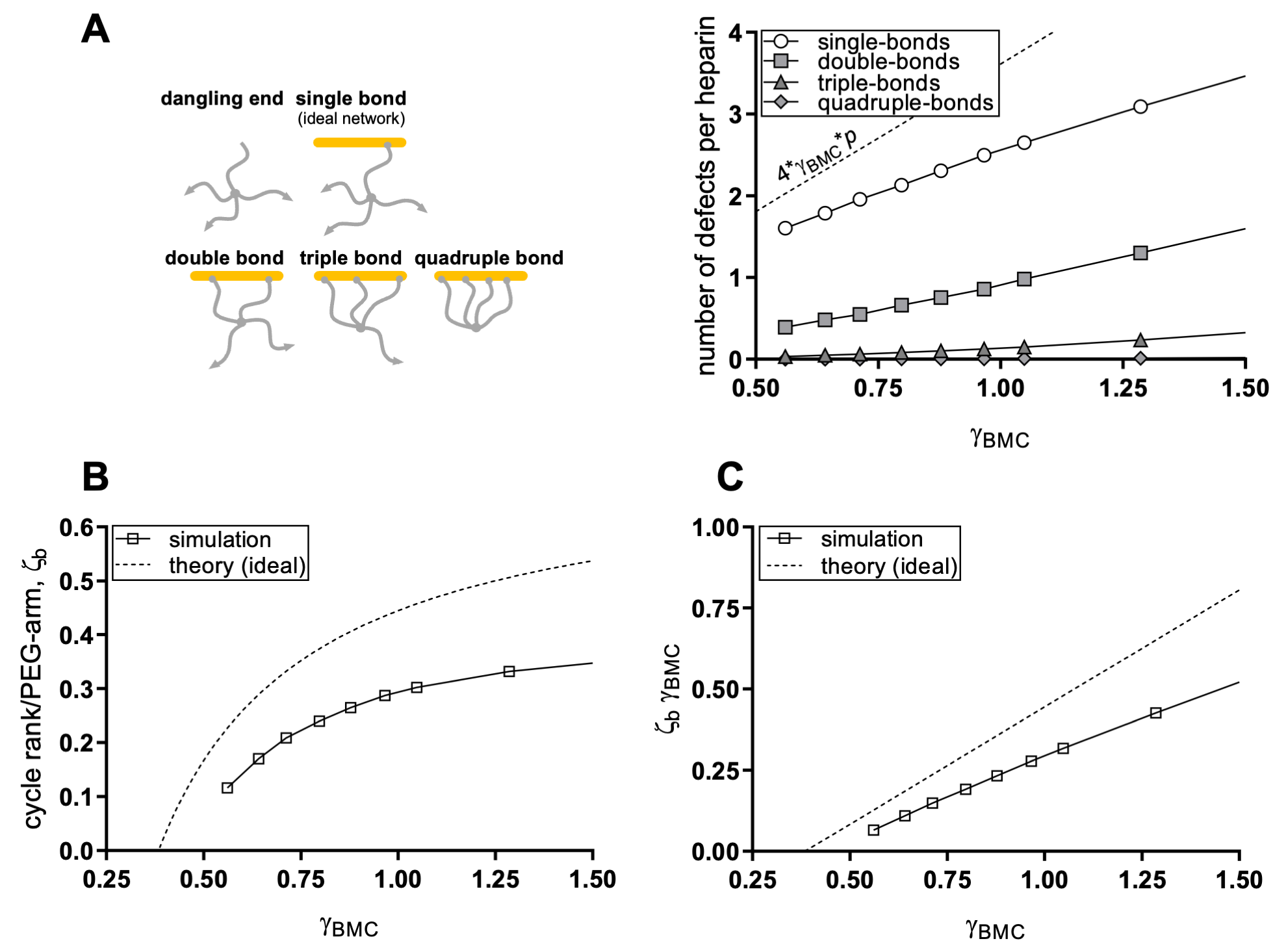 |
| --- |
| Figure S5. Simulation of starPEG-heparin network defect structures and stability. A: Simulation of primitive starPEG defect structures in the hydrogel network (here: BMC) evolving during the cross-linking process in a good solvent (e.g. PBS). Primitive defects of starPEG are schematically shown in the left panel, which are non-reacted starPEG arms that are not participating in the network structure (dangling ends) and multiple starPEG-arms (double, triple, quadruple bonds) sharing the same heparin, in contrast to the ideal network structure with elastic active strands (single bonds). The arrows denote connections to the network and to other heparins. The graph shows the total number of primitive defects per heparin molecule as a function of starPEG to heparin molar ratio *γ*_BMC_ in the BMC, with assuming an extent of reaction *p* = 0.9, similar to the experimental conditions. The dotted line represents the overall heparin-functionality (*f*HEP,BMC = 4*γ*_BMC_ · *p*) at *p* = 0.9. B: Cycle rank per strand *ζ*_b_ as function of molar ratio *γ*_BMC_ in comparison to the ideal defect-free case. C: Simulation of the stability of starPEG-heparin hydrogel networks, expressed in scaled cycle rank per strand *ζ*_b_ ·*γ*_BMC_ (measure for connectivity) as a function of *γ*_BMC_. The dotted line represents the theoretical ideal value (no defects) at *p* = 0.9. |

The molar ratio in the biggest molecule cluster *γ*_BMC_ only represents an indirect measure of stability and elasticity of the imperfect network, thus the cycle rank ζ was investigated, which relates to the pre-factor $\frac{“ f-2”}{f}$ in the phantom networkmodel [11]. Following the idea by Scanlan [12], Case [13], and Flory [11] a graph theoretical approach is used to define independent circuits, which form the polymer network. Here, the network is divided into active junctions *μ*_SC_ as crosslinking points with at least three independent connections into the polymer network and active chains *ν*_SC_ as polymer strands terminated by two active junctions. Flory generalized this concept in terminology of graph theory by considering *ν*_F_ edges and *μ*_F_ vertices in the network providing the invariant cycle rank ζ as the minimum number of scissions to reduce the graph into a spanning tree. The resulting cycle rank in both cases is invariant and can be transformed into each other by [11]

$$\zeta= \nu_{F}- \mu_{F}+1= \nu_{SC}- \mu_{SC}+1, (4)$$

for an arbitrary network.

For the ideal cross-linking process of the heparin-starPEG structure without defects, the functionalities are given by *f*_PEG_ = 4 · *p* and *f*_HEP_ = 4*γ* · *p* at the extent of reaction *p*, respectively. The average functionality of all crosslinks at a conversion *p* is obtained by [7]

$$f= \frac{8\gamma*p}{1+ \gamma}. (5)$$

The prefactor for the phantom network model is given by [7]

$$\frac{f-2}{f}= \left( 1-\frac{1}{4p} \right)*\left( 1-\frac{1}{\left( 4p-1 \right)*\gamma} \right)= g_{0}* \left( 1-\frac{\gamma_{C}}{\gamma} \right). (6)$$

relating the factor *g*_0_ and critical molar ratio *γ*_C_ to the extent of reaction *p* and molar ratio *γ*.

In the ideal case there are 4*p*·*n*_PEG_ edges connecting *n*_PEG+nHEP_ vertices spanning the network with cyclic rank

$$\zeta=4p* n_{PEG}- n_{PEG}* \left( 1+\frac{1}{\gamma} \right)+1. (7)$$

The cyclic rank per strand *ζ*_b_ in the ideal heparin-starPEG network is given by

$$\zeta_{B}= \frac{\zeta}{4p* n_{PEG}} \cong\left( 1-\frac{1}{4p} \right)*\left( 1-\frac{1}{\left( 4p-1 \right)*\gamma} \right)=\frac{f-2}{f}. (8)$$

relating the average functionality of crosslinks to the graph theory invariant.

The cycle rank in the simulation *ζ* is quantified by transforming the BMC of the network into graph with *μ*_SC_ active junctions and *ν*_SC_ active chains in the biggest molecule cluster as depicted in Figure S6. In the first step, all unreacted PEG chains are removed and the multiple bond defects are transformed into single bonds to account for the reduced effect on the elasticity and the constrained fluctuations of the junctions. In the second step, all remaining bifunctional junctions are eliminated, the remaining edges are merged, and all dangling material with only one connection to the BMC is removed recursively. As result, the BMC is transformed into a graph in terms of the Scanlan-Case theory with a cycle rank ζ = *ν*_SC_ − *μ*_SC_ +1. The reduced cycle rank per strand *ζ*_b_ in the BMC is then defined in accordance with Eq. (8) yielding

$$\zeta_{b}* \gamma_{BMC}= \frac{\zeta}{4p* n_{PEG}}* \gamma_{BMC}= g_{0}* \left( \gamma_{BMC}- \gamma_{C} \right). (9)$$

Fig. S5C shows *ζ*_b_ · *γ*_BMC_ obtained in the simulation at an extent of reaction *p* = 0.90 in comparison of the ideal prediction with *g*_0_ ≃ 0.72 and *γ*_C_ = 0.385. A linear fit with Eq. (9) yields for the simulation data *g*_0,fit_ ≃ 0.48 and *γ*_C,fit_ ≃ 0.41. The deviations to the ideal behavior are direct consequences of the underlying multiple bond defect structure, but despite of the imperfections the network gets more stable as the molar ratio *γ* increases.

| 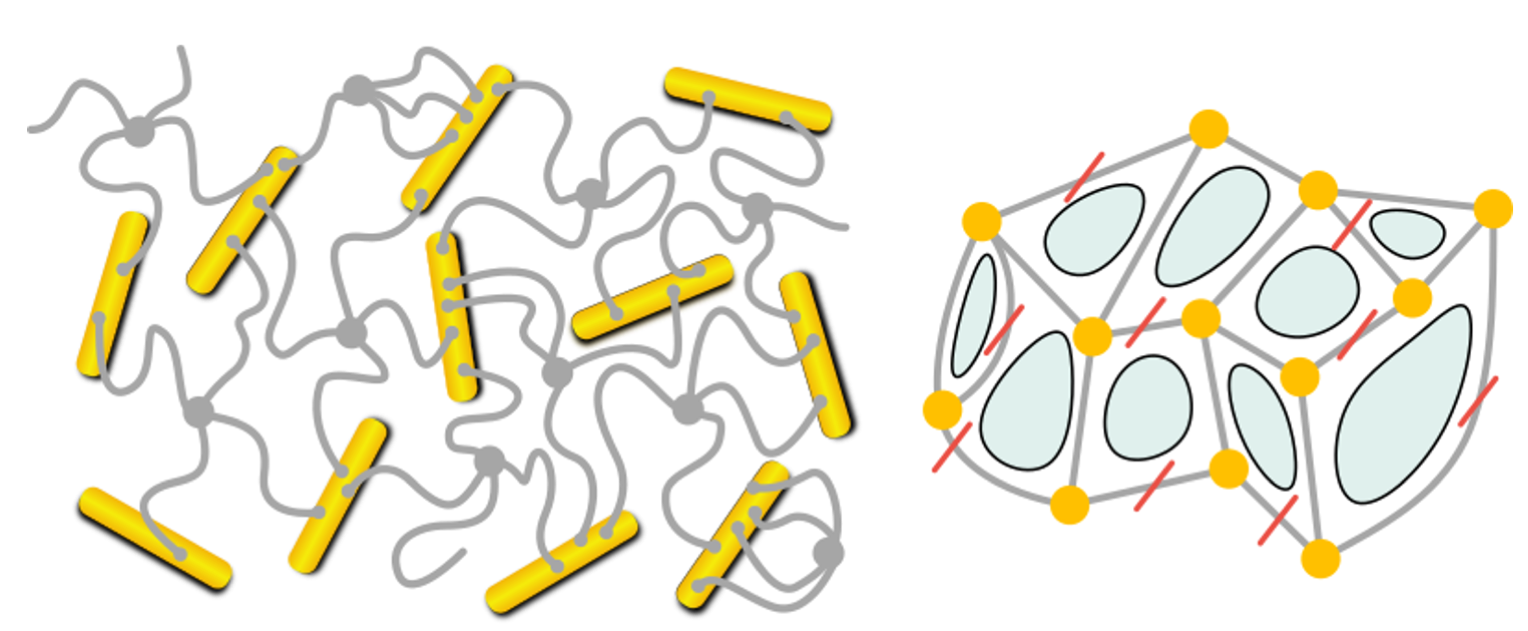 |
| --- |
| Figure S6. Scheme for transforming the starPEG-heparin network to obtain the cycle rank of the network. Shown is a representative starPEG-heparin network (left) and the corresponding transformed graph (right). The cycle rank network has *µ*_F_ = 21 vertices of tetra-functional *n*_PEG,c_ (gray circles) and *f*_HEP_-functional *n*_HEP,c_ (orange rods) connected with *ν*_F_ = 29 reduced edges. The cycle rank for the BMC is given by *ζ*_F_ = *ν*_F_ − *µ*_F_ +1 = 9 and *ζ*_SC_ = *ν*_SC_ − *µ*_SC_ +1 = 9 introducing *µ*_SC_ = 12 active junctions (orange circles) and *ν*_SC_ = 20 active chains (gray lines). The cleavage of *ζ* = *ζ*_F_ = *ζ*_SC_ = 9 edges (red lines) is needed to transform the graph with cycles (green area) into a spanning tree without cycles. |

[1] C. Carmesin, K. Kremer, The Bond Fluctuation Method: A New Effective Algorithm for the Dynamics of Polymers in All Spatial Dimensions, Macromolecules 21 (1988) 2819–2823. https://doi.org/10.1021/MA00187A030/ASSET/MA00187A030.FP.PNG_V03.

[2] H.P. Deutsch, K. Binder, Interdiffusion and self‐diffusion in polymer mixtures: A Monte Carlo study, J Chem Phys 94 (1991) 2294–2304. https://doi.org/10.1063/1.459901.

[3] T. Müller, M. Wengenmayr, R. Dockhorn, H. Rabbel, M. Knespel, A. Checkervarty, V. Sinapius, Y. Guo, M. Werner, LeMonADE-project/LeMonADE: LeMonADE v2.2.2, (n.d.). https://doi.org/10.5281/ZENODO.5061542.

[4] F. Oesterhelt, M. Rief, H.E. Gaub, Single molecule force  spectroscopy by AFM indicates helical structure of poly(ethylene-glycol) in water, New J Phys 1 (1999) 6. https://doi.org/10.1088/1367-2630/1/1/006.

[5] J.P. Wittmer, P. Beckrich, H. Meyer, A. Cavallo, A. Johner, J. Baschnagel, Intramolecular long-range correlations in polymer melts: The segmental size distribution and its moments, Phys Rev E Stat Nonlin Soft Matter Phys 76 (2007) 011803. https://doi.org/10.1103/PHYSREVE.76.011803/FIGURES/14/THUMBNAIL.

[6] G. Pavlov, S. Finet, K. Tatarenko, E. Korneeva, C. Ebel, Conformation of heparin studied with macromolecular hydrodynamic methods and X-ray scattering, European Biophysics Journal 32 (2003) 437–449. https://doi.org/10.1007/S00249-003-0316-9.

[7] J.U. Sommer, R. Dockhorn, P.B. Welzel, U. Freudenberg, C. Werner, Swelling equilibrium of a binary polymer gel, Macromolecules 44 (2011) 981–986. https://doi.org/10.1021/MA1019363/ASSET/IMAGES/MEDIUM/MA-2010-019363_0004.GIF.

[8] T. Sakai, T. Matsunaga, Y. Yamamoto, C. Ito, R. Yoshida, S. Suzuki, N. Sasaki, M. Shibayama, U. Il Chung, Design and fabrication of a high-strength hydrogel with ideally homogeneous network structure from tetrahedron-like macromonomers, Macromolecules 41 (2008) 5379–5384. https://doi.org/10.1021/MA800476X/ASSET/IMAGES/LARGE/MA-2008-00476X_0010.JPEG.

[9] A. Hoffmann, J.U. Sommer, A. Blumen, Statics and dynamics of dense copolymer melts: A Monte Carlo simulation study, J Chem Phys 106 (1997) 6709–6721. https://doi.org/10.1063/1.473668.

[10] C. Jentzsch, M. Werner, J.U. Sommer, Single polymer chains in poor solvent: Using the bond fluctuation method with explicit solvent, Journal of Chemical Physics 138 (2013). https://doi.org/10.1063/1.4792201/73121.

[11] P.J. Flory, Elastic Activity of Imperfect Networks, Macromolecules 15 (1982) 99–100. https://doi.org/10.1021/MA00229A019/ASSET/MA00229A019.FP.PNG_V03.

[12] J. Scanlan, The effect of network flaws on the elastic properties of vulcanizates, Journal of Polymer Science 43 (1960) 501–508. https://doi.org/10.1002/POL.1960.1204314219.

[13] L.C. Case, Branching in polymers. I. Network defects, Journal of Polymer Science 45 (1960) 397–404. https://doi.org/10.1002/POL.1960.1204514609.
